# Mechanically induced dysregulation of calcium homeostasis: a molecular linchpin in asthmatic pathogenesis

**DOI:** 10.1186/s40001-025-03715-9

**Published:** 2025-12-23

**Authors:** Sihao Zhu, Xinxin Xing, Jia Zheng, Hai Wang

**Affiliations:** 1 Heilongjiang University of Chinese Medicine, Harbin, 150040 Heilongjiang China; 2 Second Department of Pediatrics, First Affiliated Hospital, Heilongjiang University of Chinese Medicine, Harbin, 150040 Heilongjiang China; 3 Third Department of Acupuncture and Moxibustion, First Affiliated Hospital, Heilongjiang University of Chinese Medicine, Harbin, 150040 Heilongjiang China

**Keywords:** Calcium signaling, Mechanotransduction, Epithelial barrier dysfunction, Airway remodeling, Piezo1

## Abstract

Asthma is a heterogeneous chronic airway disease characterized by inflammation, hyperresponsiveness, mucus hypersecretion, and remodeling. Emerging evidence highlights calcium homeostasis imbalance as a central molecular hub integrating mechanical stress with immune-inflammatory responses. This review synthesizes recent mechanistic insights into how dysregulated calcium signaling—via transient receptor potential (TRP) channels (e.g., TRPV1, TRPA1), store-operated calcium entry (SOCE), L-type calcium channels (LTCCs), and mechanosensitive Piezo1—drives key asthma phenotypes. Calcium fluctuations triggered by mechanical stimulation disrupt the epithelial barrier. This process is achieved through activating calcium protease, which degrades E-cadherin and occludin, a tight junction protein. Simultaneously, it enhances the release of Th2-type cytokines (e.g., IL-4 and IL-13) and sustains the pathological state of mucosal cell proliferation via the TMEM16A channel. In airway smooth muscle, calcium dyshomeostasis enhances contractility via the myosin light-chain kinase (MLCK)/RhoA-ROCK axis and SOCE hyperactivation, while Piezo1-mediated mechano transduction exacerbates extracellular matrix (ECM) deposition and remodeling. We propose a bidirectional “calcium-barrier-inflammation vicious cycle” where mechanical stress and cytokines synergize to sustain pathology. Interventions targeting calcium-regulated nodes (such as STIM1-Orai1 and Piezo1) may provide a new direction for asthma treatment beyond traditional anti-inflammatory strategies.

## Introduction

Asthma is a heterogeneous disease triggered by diverse factors including pollen, mold, viruses, and emotional changes [1, 2]. It is characterized by pathological hallmarks including inflammation factor-driven airway hyperresponsiveness (AHR), distinct types of chronic airway inflammation (Type 2-high and Type 2-low), airway mucus hypersecretion (AMH), and airway remodeling, often accompanied by reversible airflow limitation [3, 4]. Ion channels serve as a fundamental nexus for signaling within asthma effector cells. They are not merely isolated factors contributing to singular pathological features, but rather act as a molecular linchpin orchestrating the entire disease course. Key channels implicated include transient receptor potential (TRP) channels, store-operated calcium entry (SOCE) Channels, calcium-activated potassium channels (IKCa and BKCa), the calcium-activated chloride channel TMEM16A, the mechanosensitive ion channel component Piezo1, and purinergic P2X receptors (P2X). These channels exhibit specific or varying degrees of expression in respiratory system-associated cells, including lung fibroblasts, airway smooth muscle cells (ASMCs), pulmonary endothelial cells, and alveolar and bronchial epithelial cells [5]. Calcium homeostasis, a core regulatory network governing cellular life processes, relies on the coordinated actions of plasma membrane calcium channels, calcium pumps, and intracellular calcium stores. Mounting evidence links disruptions in calcium homeostasis (calcium dysregulation) not only to tumorigenesis [6] and cardiovascular diseases [7–9], but also to a critical role in the pathogenesis and progression of bronchial asthma.

### The biological essence and regulatory hierarchy of calcium homeostasis

The biological essence of calcium homeostasis lies in maintaining a strictly controlled dynamic equilibrium of calcium concentration across intracellular and extracellular compartments [10]. This critical equilibrium is rigorously maintained by calcium buffering systems [11], and is fundamental to essential cellular functions, including the regulation of gene expression, muscle contraction, neuronal transmission, and enzymatic activity [12–14]. Under resting conditions, the intracellular calcium concentration ([Ca^2^⁺]i) (~ 50–100 nM) is markedly lower than the extracellular level (~ 1–2 mM). This substantial gradient is actively maintained through the coordinated actions of intracellular calcium stores, ion channels, pumps (e.g., plasma membrane Ca^2^⁺-ATPase, sarco/endoplasmic reticulum Ca^2^⁺-ATPase—SERCA), exchangers (e.g., Na⁺/Ca^2^⁺ exchanger—NCX), and organelles such as the endoplasmic reticulum (ER) and mitochondria [11].Upon cellular stimulation (e.g., depolarization or ligand binding), [Ca^2^⁺]i levels undergo transient elevations (e.g., via voltage-gated calcium channels (VGCCs) or inositol trisphosphate receptor (IP₃R)-mediated release). These increases are followed by rapid restoration to baseline, generating characteristic “calcium transients”.

The regulation of cellular calcium homeostasis represents a highly complex and multi-tiered process. It involves the orchestrated interplay of multiple organelles, including the plasma membrane, ER, and mitochondria, and is achieved through intricate regulatory networks comprising diverse proteins, channels, and signaling pathways. This concerted action is fundamental to maintaining dynamic equilibrium and constitutes a major component of the cellular calcium buffering system. Mitochondria regulate calcium fluxes primarily through the mitochondrial calcium uniporter (MCU) for uptake, and the mitochondrial Na⁺/Ca^2^⁺ exchanger (NCLX) and Ca^2^⁺/H⁺ exchanger (CHE) for efflux. Collectively, these transport systems maintain the matrix calcium concentration, thereby modulating key metabolic processes such as the tricarboxylic acid (TCA) cycle and ATP synthesis [15]. The ER serves as the primary intracellular calcium store. Upon activation of phospholipase C (PLC), PLC catalyzes the hydrolysis of phosphatidylinositol 4,5-bisphosphate (PIP₂), generating inositol 1,4,5-trisphosphate (IP₃). IP₃ binds to IP₃ receptors (IP₃Rs) on the ER membrane, triggering calcium release from the ER lumen into the cytosol and elevating [Ca^2^⁺]i. Ryanodine receptors (RyRs) can further amplify this calcium release in response to calcium signals (calcium-induced calcium release—CICR), Depletion of ER calcium stores induces a conformational change (oligomerization) in stromal interaction molecule (STIM) proteins. Oligomerized STIM proteins subsequently translocate to ER-plasma membrane junctional regions. There, they interact with Orai proteins to form the STIM-Orai complex, which mediates SOCE—the influx of extracellular Ca^2^⁺ through Orai channels, replenishing ER stores. The SERCA pump actively transports Ca^2^⁺ from the cytosol against its concentration gradient into the ER lumen, utilizing ATP hydrolysis. This process maintains the high luminal Ca^2^⁺ concentration within the ER and is critical for overall cellular calcium homeostasis. SERCA is therefore a key regulator of ER calcium balance.Furthermore, the ER exerts regulatory functions through its dynamic interactions with other organelles, including mitochondria, lysosomes, and the plasma membrane (e.g., via membrane contact sites—MCSs). At the plasma membrane, calcium influx is mediated by channels such as: VGCC (e.g., L-, T-, N-types), receptor-operated or second messenger-gated channels (e.g., TRP family members), and SOCE channels (e.g., the STIM1-Orai1 complex, potentially involving TRPC1). Conversely, Ca^2^⁺ efflux is facilitated by the Na⁺/Ca^2^⁺ exchanger (NCX) and the plasma membrane Ca^2^⁺-ATPase (PMCA). This exquisite regulation of calcium fluxes is paramount for cell survival, metabolism, and function, and its dysregulation underlies various pathological conditions.

The mechanisms governing cellular Ca^2^⁺ transport rely on the sophisticated regulation by organelles including the plasma membrane, ER, and mitochondria. This encompasses key processes such as SOCE, VGCC-mediated influx, and second messenger-triggered release (e.g., via IP₃). Additionally, calcium-binding proteins, notably calmodulin (CaM), play crucial modulatory roles in these pathways. This regulatory process involves the orchestrated interplay of diverse components: calcium channels (including voltage-gated, ligand-gated, and store-operated types), calcium pumps (such as the SERCA and plasma membrane Ca^2^⁺-ATPase—PMCA), buffering proteins (e.g., CaM), and signaling molecules (e.g., IP₃ and diacylglycerol—DAG). Furthermore, extracellular hormones, such as parathyroid hormone (PTH) and calcitonin, contribute to the systemic regulation of calcium homeostasis. Fundamentally, the specificity of calcium signaling is achieved through spatiotemporal encoding mechanisms: spatial compartmentalization (e.g., via distinct Ca^2^⁺ stores within the ER and mitochondria) and temporal encoding (e.g., through Ca^2^⁺ oscillations) [16]

### The role of calcium channels in asthma: TRPs, SOCE, LTCC, and Piezo1

#### TRP channels (TRPV1/TRPA1/TRPV4)

TRP channels are a class of non-selective cation channels that can sense various stimuli such as temperature, chemicals, and mechanical forces. They consist of six transmembrane regions forming the core structure of ion channels. The varying lengths of intracellular ends in different TRP channels determine their functional differences [17]. In the respiratory tract, TRPV1, TRPA1, and TRPV4 are widely distributed. When activated, these receptors allow calcium, sodium, and magnesium ions to enter cells, altering intracellular conditions. They also activate signaling pathways such as MAPK, TGF-β, and NF-κB, which play roles in inflammatory responses and tissue remodeling [18]. The TRPA subfamily governs chemical sensing and neurogenic inflammation. TRPA1 can sense environmental stimuli, temperature changes and osmotic pressure [19, 20], helps maintain the epithelial barrier [21]. In patients with asthma and COPD, TRPA1 expression is abnormal and closely related to epithelial injury [5], when activated, it triggers the release of neuropeptides that trigger airway constriction, increased mucus secretion and hyperreactivity [22]. In ASMCs, TRPA1 acts as an active driver of asthmatic inflammation and AHR [23]. TRPV channels integrate thermal [24] osmotic [25] and mechanical cues [26], and cooperate with TRPC channels to propagate calcium signals. Infections (such as RSV) increase the expression of TRPV1 in the bronchial epithelium, making it more sensitive to stimulation, enhancing Ca2⁺ influx, and promoting inflammation and hyperreactivity [27]. In mice with TRPV1 deficiency, airway inflammation, goblet cell proliferation and collagen deposition were significantly reduced [28, 29]. Polymorphisms in TRPA1 and TRPV1 differentially influence asthma control in paediatric populations [20]. Both high and low humidity impair epithelial function and mucociliary clearance. Co-exposure to high humidity and nitrogen dioxide (NO₂) exacerbates histopathological alterations and AHR in asthmatic mice, up-regulating allergic and inflammatory markers via oxidative stress, inflammatory cascades and immune-cell activation, and concomitantly elevating TRPA1, TRPV1 and TRPV4 expression [30, 31]. After TRPV4 activation, airway remodeling is promoted by NOX4 and TGF-β1 signals [32]. It can also activate ADAM10 through calcium signaling, shear E-cadherin, disrupt epithelial junctions, increase permeability, and promote epithelial-mesenchymal transition (EMT) and inflammatory processes [33]. TRPV4 is expressed in alveolar type II (AT2) cells and affects epithelial cell damage repair and EMT, promoting inflammation [34]. Cold-air challenge evokes airway constriction by activating mechano- and thermo-sensitive TRP channels. Arachidonic acid and its metabolites sustain Ca^2^⁺ influx, thereby maintaining [Ca^2^⁺]i elevation, triggering cascades that amplify inflammation, AHR and collagen deposition—thereby promoting airway remodelling. These mechanisms provide a molecular framework for asthma exacerbations triggered by cold or heat exposure [35, 36].

#### SOCE channels

The ER is sensitive to mechanical forces. The channels on its membrane can sense external forces, regulate the release and recovery of calcium ions, and affect cellular signals and stress responses [37, 38]. ER calcium homeostasis imbalance induces inflammation and tissue remodeling and is involved in the pathogenesis of asthma [39]. Extracellular cues, mechanical stress and oxidative stimuli activate phospholipase C (PLC)/IP₃ signalling, prompting IP₃ receptor-mediated Ca^2^⁺ efflux from the ER and a concomitant rise in [Ca^2^⁺]i—a pivotal event in mucus secretion [40]. When ER calcium storage decreases, STIM1 binds to Orai1 and activates the Ca^2^⁺ release-activated Ca^2^⁺ (CRAC) channel [41], triggering SOCE,and causing sustained Ca^2^⁺ influx. Oxidative stress or ER stress also inhibits SERCA pumps, further enhancing the STIM1-Orai1 signaling pathway. Moreover, mechanosensitive ion channels such as TRPV1 are expressed in the ER membrane and translate ER mechanical strain into calcium signals [42]. By integrating calcium signalling, inflammatory cascades, immune modulation and airway remodelling, the ER-Ca^2^⁺ signaling machinery emerges as a central molecular nexus that links disparate pathogenic pathways in asthma.

Early work on mesenchymal stem cells demonstrated that cyclic stretch transmits mechanical force to intracellular compartments, evoking delayed Ca^2^⁺ release from the ER [43]. Using a direct mechanical probe on the ER and the Ca^2^⁺ indicator GCaMP6. Song and colleagues observed a rapid (~ 10–20 s) fluorescence increase that peaked shortly after stimulus onset, confirming that mechanically evoked [Ca^2^⁺]i transients originate from ER stores [44]. Prior studies have established that extracellular mechanical forces are relayed to intracellular organelles via the cytoskeleton [43]. In a complementary study on airway epithelial cells(AECs) subjected to cyclic stretch, confocal imaging revealed that pre-treatment with the Piezo1 antagonist GsMTx4 or the Ca^2^⁺ chelator EGTA abolished the mechanically induced Ca^2^⁺ influx, yielding signals indistinguishable from static controls. These data convincingly demonstrate that stretch-induced Ca^2^⁺ influx is predominantly Piezo1-dependent [45], yet they indirectly imply negligible ER Ca^2^⁺ release under the same conditions—a finding that appears to conflict with earlier observations. Such discrepancies may reflect cell-type-specific sensitivities to mechanical stimulation. Follow-up experiments employing ER-targeted Ca^2^⁺ indicators are therefore warranted to determine (i) whether mechanical stretch elicits ER Ca^2^⁺ release in AECs and (ii) the precise temporal relationship between ER Ca^2^⁺ mobilization and extracellular Ca^2^⁺ entry.

#### L-type calcium channels (LTCCs)

LTCCs, a subtype of high-voltage-activated calcium channels, play a central role in regulating ASM contraction and amplifying inflammatory responses, both of which are key drivers of asthma pathogenesis. LTCCs are abundantly expressed in human ASMCs. Membrane depolarization triggers their opening, leading to rapid Ca^2^⁺ influx, activation of myosin light-chain kinase (MLCK), and subsequent ASM contraction. Sustained or aberrant LTCC activation is increasingly recognized as a core molecular mechanism underlying AHR [46]. Although mechanical forces—such as shear stress, stretch, or extracellular matrix stiffness—do not directly gate LTCCs, they indirectly modulate channel activity by altering membrane biophysical properties, including tension, curvature, and fluidity. This modulation occurs via two main pathways [47]: changes in membrane potential that influence LTCC gating, and M2 muscarinic receptor (M2R)-mediated inhibition of SERCA, which reduces intracellular Ca^2^⁺ buffering and enhances calcium signaling. In summary, LTCC activation serves as a key integrator of electromechanical coupling and receptor-mediated signaling, translating mechanical microenvironmental cues into precise spatiotemporal regulation of channel activity and expression [48].

LTCCs play a pivotal role in the pathogenesis of asthma. Their activation contributes to the exacerbation of asthmatic pathologies not only by modulating the contraction of ASMCs, but also through interactions with inflammatory responses, mucus hypersecretion, and airway remodeling processes. Aberrant activation of LTCCs further elevates [Ca^2^⁺]i, resulting in hypercontraction of ASMCs and potentiating inflammatory responses. Studies demonstrate that interleukin-25 (IL-25) significantly upregulates both the transcriptional and protein levels of CaV1.2 (Cacna1c) in primary cultured murine tracheal smooth muscle cells [49], CaV1.2 serves as a critical effector molecule mediating IL-25-induced enhancement of ASMC contraction and promotion of AHR. The expression and function of CaV1.2 are regulated by IL-25 via a specific signaling pathway: IL-17RA/IL-17RB → TAK1 → TPL2 → MEK1/2 → ERK1/2 → AP-1. LTCC activation is intimately linked to enhanced T cell function. Specifically, upregulated expression of LTCCs (e.g., CaV1.2 and CaV1.3) in T helper 2 (Th2) cells augments tyrosine kinase activity within these cells, thereby promoting the expression of Th2 cytokines (e.g., IL-4, IL-5, IL-13) [50]. PP121, an inhibitor of tyrosine and phosphoinositide kinases, attenuates AHR by relaxing pre-contracted murine tracheal rings (mTRs) through blockade of multiple ion channels, including LTCCs. Furthermore, it mitigates systemic inflammation and mucus secretion by downregulating inflammatory cytokines (e.g., IL-4, IL-5, TNF-α), mucins (MUC5AC, MUC5B), and the MAPK/Akt signaling pathway. In mast cells, activation of Mas-related G protein-coupled receptor X2 (MrgPRX2/MrgX2) by ligands such as allergens elicits downstream effects—mast cell degranulation and release of inflammatory mediators—via the CaMKII/PKC/CaV1.2 pathway [51]. LTCC activation-induced membrane depolarization activates TMEM16A channels. This activation promotes chloride and fluid secretion, culminating in mucus hypersecretion [52]. TMEM16A achieves this by upregulating its own expression in goblet cells, directly facilitating mucus exocytosis, and modulating the expression of inflammation-associated transcription factors such as SPDEF. Ca^2+^ influx further contributes indirectly to airway remodeling and structural alterations by influencing goblet cell hyperplasia through modulation of apoptosis and cell cycle regulation.

LTCCs contribute to the exacerbation of asthma through multiple mechanisms, including inflammation amplification, AHR, mucus hypersecretion, and airway remodeling. This involvement is mediated by the dysregulation of Ca^2+^ influx, the aberrant activation of intracellular calcium signaling, and the cascade activation of downstream pathways, which collectively exacerbate the pathological processes of asthma.

#### Piezo-type mechanosensitive ion channel component 1 (Piezo1)

Piezo1 is a mechanosensitive cation channel abundantly expressed in airway epithelium, endothelium and pulmonary macrophages [53]. Its trimeric clover-shaped structure comprises three subunits of 38 transmembrane helices each that encircle a central Ca^2^⁺-selective pore [54, 55]. Forces transmitted via membrane lipids or ECM–cytoskeletal tethers elicit millisecond-scale conformational opening, allowing rapid Ca^2^⁺ influx [54, 56–58]. Upon application of mechanical forces (e.g., shear stress, membrane stretch) to the plasma membrane, conformational changes in the Piezo1 peripheral domains induce a transition from the closed to the open state. This activation occurs within milliseconds, permitting cation influx (e.g., Ca^2^⁺) into the cell [56], and Yoda1-evoked [Ca^2^⁺]i rises in ASMCs are abolished by EGTA, confirming extracellular Ca^2^⁺ as the immediate source [45, 59]. This calcium signaling propagates downstream cascades that couple mechanical stress to asthma-related inflammation, remodeling and hyperresponsiveness.

In human pulmonary microvascular endothelial cells (HPMECs), researchers stimulated the cells using Yoda1, a selective activator of Piezo1, and observed a time-dependent increase in ROCK1 mRNA expression. This indicates that Piezo1 activates the RhoA/ROCK1 signaling pathway. Conversely, when Piezo1 function was blocked using Piezo1-siRNA or its inhibitor GSMTx4, these effects were eliminated. These findings confirm that Piezo1 participates in mechanical stretch-induced cellular injury through activation of the RhoA/ROCK1 signaling pathway and may play a role in the development of ventilator-induced lung injury (VILI) [60]. In primary human small airway epithelial cells (HSAECs) grown at an air-liquid interface, a single 10 cm H_2_O extra pressure for 12 h triggered a sustained [Ca^2+^] rise; this Piezo1-mediated Ca^2+^ signal reduced occludin, ZO-1 and claudin-18 abundance and doubled paracellular flux. All changes were reversed by the Piezo1 blocker GsMTx-4 (2 μM), mechanistically linking elevated airway pressure  → Piezo1 opening  → Ca^2+^-dependent tight-junction degradation  → barrier leak [61]. Additional work indicates that Piezo1 may impair nasal epithelial barrier function by down-regulating tight junctions through Ca^2+^-ERK1/2 signaling [62]. The functional outcome of Piezo1 activation is context-dependent: in pulmonary group 2 innate lymphoid cells (ILC2s), IL-33 up-regulates its expression; genetic deletion exaggerates ILC2 activity and AHR, whereas the agonist Yoda1 induces KLF2 to suppress NF-κB, restrains mitochondrial respiration in ILC2s, and attenuates human ILC2-driven AHR in a humanized mouse model [63]. Inhibitors such as GsMTx4 relieve membrane curvature stress, causing the channel "blades" to collapse and thus blocking Piezo1 activity, offering a novel therapeutic strategy for asthma [55]. The key calcium signaling nodes discussed above, their roles in different airway cell types under mechanical stress, and the corresponding experimental evidence are summarized in Table 1.

**Table 1:** Summary of evidence for mechanical-calcium signaling pathways in different cell types

Calcium nodes	Cell type	Pathophenotype/function	Key mechanical stresses	Evidence level	Causal reading certificate
Piezo1	Airway epithelial cells	Barrier disruption (degradation of tight junctions) and increased permeability	High airway pressure, traction	In vitro, animal models	Pharmacological inhibition (GsMTx4), gene knockdown
TRPV4		Barrier disruption (E-cadherin detachment), inflammatory response	Tension, ECM, shear stress	In vitro, animal models	Pharmacological inhibition, gene knockout
SOCE (STIM1/Orai1)		Mucin secretion (MUC5AC), release of inflammatory factors	Tension, cell compression	In vitro, animal models	Pharmacological inhibition (CM2489, etc.), genetic manipulation
Piezo1	airway smooth muscle cells	Increased contraction, cell migration, airway remodeling	Tension, compression	In vitro, animal models	Pharmacological activation/inhibition (Yoda1, GsMTx4)
SOCE (STIM1/Orai1)		Constriction, airway hyperreactivity (AHR), proliferation	Tension (indirect through membrane potential/GPCR)	Extracellular, animal and human tissue	Pharmacological inhibition, gene knockdown (human primary cells)
TRPC3		Ca^2^⁺ influx, airway remodeling	Stretch	In vitro, animal models	Overexpression/interference model
LTCC (CaV1.2)		Contractile enhancement, AHR, inflammatory response	Stretch (indirect through membrane potential)	In vitro, animal models	Pharmacological inhibition, signal pathway blockade
Piezo1	lung fibroblast	Fibroblast-muscle fibroblast conversion (FMT), ECM production	Cyclic stretch	Extracellular cell model	Pharmacological inhibition, siRNA knockdown
Piezo1	ILC2	Inhibiting ILC2 function and relieving AHR	Not yet clear (induced by IL-33)	Animal model	Genetic deficiency, pharmacological activation (Yoda1)

The contents of this table are only the relevant evidence that the author has consulted, which may not cover all the research on the above content, and can provide readers with a certain direction for reference

### Mechanisms of interaction between airway epithelial barrier disruption and aberrant calcium signaling

#### Significance of the airway epithelial barrier and triggers of its disruption

The airway epithelial barrier safeguards homeostasis through spatially organized E-cadherin, occludin and ZO-1 junctions that double as innate immune hubs [64–67]. When these proteins are mutated (FLG, PCDH1, GSDMB) or environmentally challenged, the fence fails: allergen proteases directly cleave occludin/ZO-1/E-cadherin or trigger PAR-2-driven Ca^2^⁺ influx and NF-κB/MAPK signaling, while rhinovirus/adenovirus internalise or proteolyse occluding [66–72]. The resulting paracellular leak seeds chronic inflammation, viral susceptibility and remodelling.

#### Calcium dysregulation: barrier breakdown and inflammatory amplification

**Fig. 1:**
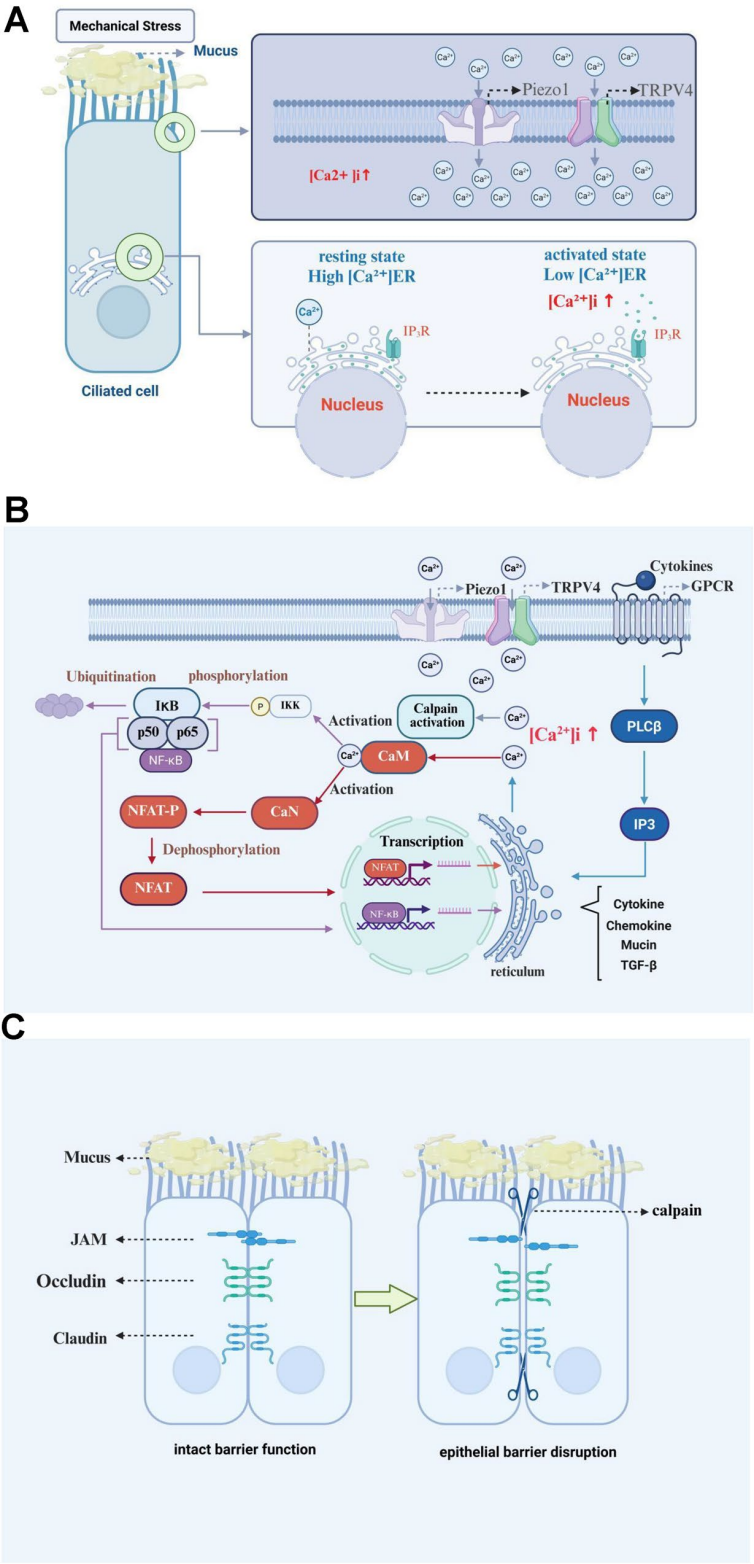
Calcium-dependent mechanisms of airway epithelial barrier dysfunction in asthma. **A** Mechanical stress (shear/stretch) gates apical Piezo1/TRPV4 and other mechanosensitive channels in ciliated cells, inducing extracellular Ca2⁺ influx; concurrent PLC-IP₃ signaling activates IP₃R and triggers ER Ca2⁺ release, rapidly elevating [Ca2⁺]i. **B** Elevated [Ca2⁺]i binds calmodulin (CaM), activating calpain and switching on the CaN-NFAT and NF-κB axes, which transcriptionally up-regulate pro-inflammatory mediators and mucins (MUC5AC), impairing barrier integrity. **C** Ca2⁺-activated calpain proteolytically cleaves tight-junction proteins Claudin, Occludin, and ZO-1, widening intercellular gaps and increasing permeability

In healthy airway epithelium, brief Ca^2^⁺ oscillations drive ciliary beating, mucus release and ZO-dependent cytoskeletal dynamics that maintain barrier integrity and wound repair [11, 73–77], Asthma disrupts this choreography: sustained Ca^2^⁺ influx—via SOCE, Piezo1/TRP stretch, or ECM-activated LTCC—activates calpains, PKC and Rho-GTPases, phosphorylating and internalising occludin, E-cadherin and ZO-1 [61, 77, 78]. The resulting paracellular leak exposes subepithelial sensors to allergens and viruses, while calcineurin-NFAT and CaMK-NF-κB cascades amplify IL-6, IL-8, TSLP and mucins that recruit eosinophils, neutrophils and Th2 cells [73, 79–82]. Excess mucus physically clogs the lumen and creates a microbial niche, whereas chronic Ca^2^⁺ loading arrests the cell cycle, skews differentiation toward goblet-cell metaplasia and triggers actomyosin remodelling that collectively cripple epithelial repair and accelerate airway wall thickening [83, 84] (Fig. 1A, B, C).

These events form a self-sustaining vicious cycle of “barrier disruption → inflammatory infiltration → further calcium dysregulation”.

#### The core significance: a self-perpetuating pathological axis

Aberrant calcium signalling and epithelial barrier loss create a self-sustaining loop that underpins asthma: environmental stressors (allergen proteases, viruses, mechanical stretch) open mechanosensitive or SOCE channels → Ca^2^⁺ overload activates calpains, PAR-2 and NF-κB → tight-junction disassembly, mucus metaplasia and cytokine release → barrier breakdown. The resulting leak facilitates irritant penetration and maintains a Ca^2^⁺-rich inflammatory milieu, perpetuating AHR and remodelling. Interrupting this circuit at nodes such as Piezo1, STIM1-Orai1, PAR-2 or calpains offers a rational therapeutic strategy.

### Calcium homeostasis imbalance mediates the molecular pathological mechanism of AMH

#### Physiology and pathophysiology of airway mucus

Airway mucus is a 93–97% water hydrogel whose viscoelastic and adhesive properties are conferred by a mesh of secreted polymeric mucins (MUC5AC/MUC5B, 3–7% wt.) and membrane-tethered mucins (MUC1/MUC4/MUC16) that project their heavily O-glycosylated extracellular domains above the epithelial surface [85–89]. This composite gel exhibits shear-thinning behaviour and an interfacial tension that allows efficient mucociliary clearance (MCC) while providing a physical and biochemical barrier against pathogens, pollutants and dehydration [86, 87]. Optimal hydration is maintained by ion-water pumps and Ca^2^⁺-activated chloride channels (TMEM16A) that fine-tune the periciliary fluid layer, ensuring that cilia beat within a low-viscosity environment [88, 89].

#### Asthma-associated mucus dysregulation

In asthma, the MUC5AC:MUC5B ratio shifts toward MUC5AC, a change that correlates with disease severity and predicts airflow obstruction [90], Type-2 cytokines (IL-4/IL-13) bind epithelial GPCRs, activating PLC/IP3-mediated Ca^2^⁺ release and store-operated Ca^2^⁺ entry; the resulting cytosolic Ca^2^⁺ peaks open TMEM16A/ANO1 channels, promoting Cl⁻ and HCO₃⁻ efflux that hydrate and expand the mucus layer [91–98]. In IL-13-stimulated Calu-3 cells, siRNA knockdown of TMEM16A blocked SPDEF upregulation and inhibited goblet cell metaplasia. Similarly, co-transfection of siT16A in N-CLCA1-induced polarized BCi-NS1 human respiratory epithelial model almost completely abolished mucus secretion. Mechanistically, IL-13 simultaneously activates the Ca^2^⁺-calcitonin-NFAT and STAT6 signaling pathways to elevate SPDEF (the primary transcription factor for MUC5AC). CLCA1, acting as a chaperone protein, anchors TMEM16A to the apical membrane, amplifying Ca^2^⁺-dependent mucus exocytosis. Thus, TMEM16A serves dual roles as both an ion channel and an essential amplifier in this signaling axis [99, 100]. Consequently, the airway lumen becomes clogged with viscous, hyper-concentrated mucus that not only obstructs airflow but also provides a nutrient-rich niche for bacterial colonisation and continual Type-2 immune activation, thereby integrating mucus overproduction with chronic inflammation, airway remodelling and neuro-immune dysregulation characteristic of severe asthma.

#### Ca^2^⁺: the master regulator of mucin gene to granule release

Airway mucus volume and composition are controlled by cytosolic Ca^2^⁺ at every level—from gene transcription to granule exocytosis. Ca^2^⁺-coupled receptors (GPCRs, P2Y purinoceptors) on goblet or submucosal gland cells initiate PLC/IP3-mediated ER store depletion; the subsequent STIM1/Orai1-driven SOCE sustains high [Ca^2^⁺]i required for prolonged secretion [101–104]. Ca^2^⁺-calcineurin-NFAT and CaMK/PKC directly transactivate the SPDEF and MUC5AC promoters, while Ca^2^⁺-activated TMEM16A furnishes the Cl⁻ and water flux that hydrates the newly secreted mucin mesh. Thus, a transient Ca^2^⁺ pulse becomes a long-lasting transcriptional and hydration signal.

#### Mechanical and inflammatory modulation of the Ca^2^⁺-mucus axis

Major gel-forming mucins (MUC5AC/MUC5B) are synthesized as high-molecular-weight monomers that undergo CysD domain-mediated Ca^2^⁺-dependent folding and disulfide-bonded polymerization within the ER-Golgi secretory pathway [105]. In healthy airways, this process generates a viscoelastic yet cleavable gel that can be readily transported by ciliary beating. In asthma, however, sustained Ca^2^⁺ influx—via SOCE, Piezo1 or TRPV4 activation—elevates intraluminal Ca^2^⁺ concentrations, which not only accelerates mucin granule exocytosis but also promotes hyperpolymerization of CysD-rich domains, yielding excessively dense mucus plugs [106]. Therapeutic disruption of these disulfide bonds with reducing agents (e.g., N-acetylcysteine) or Ca^2^⁺ chelators reverses the hyperviscous state, restoring mucociliary clearance in allergic airway disease models [107]. Additionally, bronchoconstriction-induced epithelial crowding mechanically activates Piezo1/TRPV4, further raising [Ca^2^⁺]i and enhancing MUC5AC expression through a calpain-NFAT axis [84]. In colon epithelial cells, Zhang et al. (2022) demonstrated that pore formation of GSDMD in the epithelium triggers early Ca^2^⁺ influx. This influx activates scinderin and promotes the P2Y2 receptor-mediated signaling pathway, thereby enhancing the docking and efflux of MUC2 mucin granules. This critical pathway is absent in GSDMD-deficient mice (Gsdmd − / −  − / − or GsdmdΔIECΔIEC) or in cells with P2Y2 signal blockage [73].

**Fig. 2:**
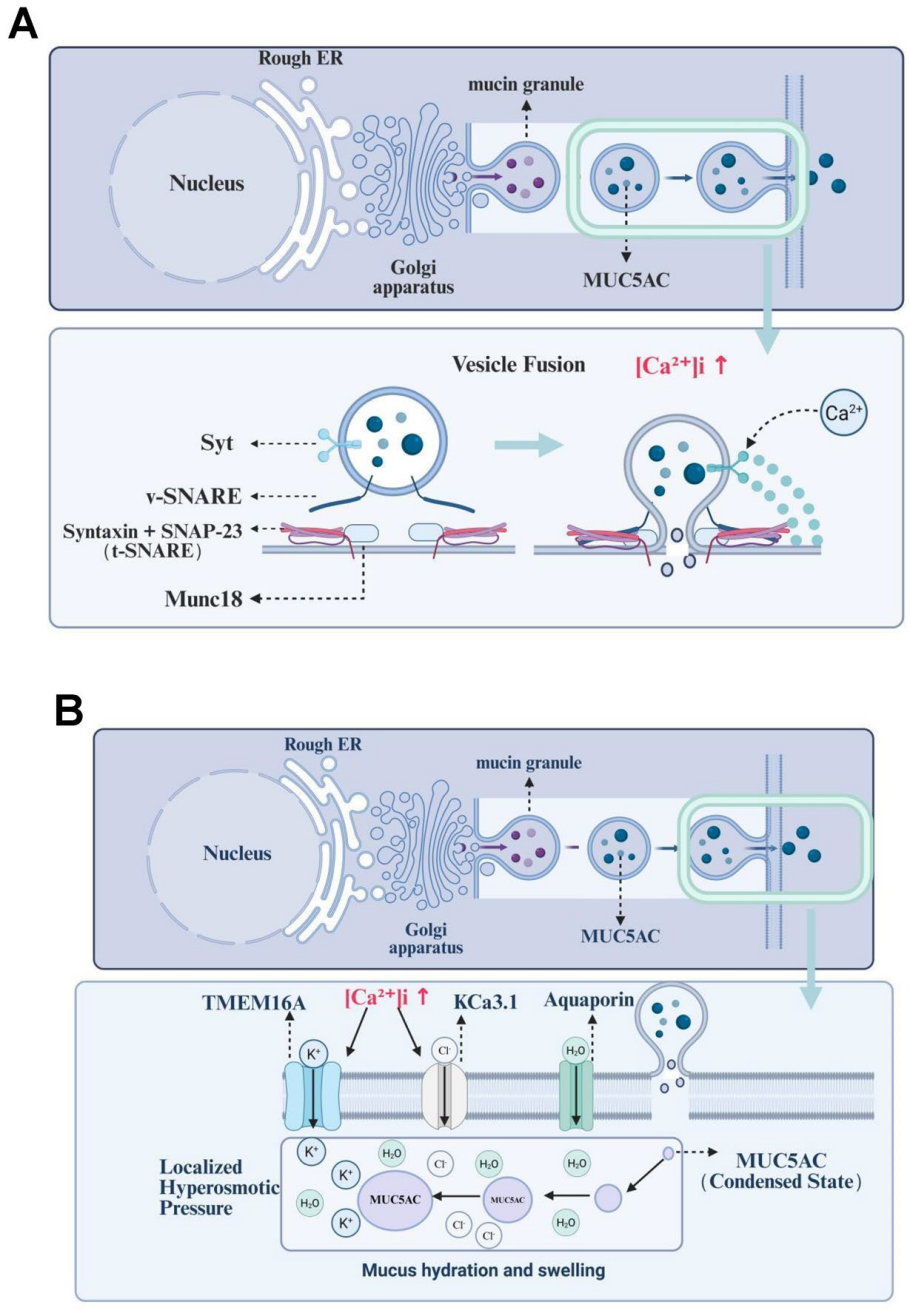
**A** Calcium-triggered vesicle-fusion pathway in hyper-secreting airway goblet cells. Hyperosmotic/shear stress evokes Ca2⁺ influx ([Ca2⁺]i↑); Ca2⁺-calmodulin enables V-SNARE–t-SNARE (Syntaxin-SNAP-23)–Munc18 complex assembly, catalyzing mucin granule fusion with the plasma membrane and MUC5AC discharge.** B** Ion-water transport coupling during mucus hydration and swelling. Sustained Ca^2^⁺ signals gate TMEM16A (Cl⁻ channel) and KCa3.1 (K⁺ channel); Cl⁻ efflux establishes an osmotic gradient, while aquaporins (AQP) drive water influx, converting condensed MUC5AC into a hydrated, expanded mucus gel

Allergen- or irritant-driven Th2 cytokines (IL-4/IL-13, histamine, tryptase) occupy H1R/CysLT1R GPCRs on goblet/gland cells, triggering PLC-mediated ER Ca^2^⁺ release and a transient cytosolic peak that primes early mucin exocytosis [108]. ER depletion promptly activates STIM1-Orai1 SOCE, providing a sustained extracellular Ca^2^⁺ influx that keeps [Ca^2^⁺]i elevated and couples mechanical or oxidative cues (stretch, ROS) to additional Ca^2^⁺ entry through TRPV4/TRPA1 [17], Elevated [Ca^2^⁺]i directly engages the vesicle fusion machinery: synaptotagmin binds Ca^2^⁺ and nucleates SNARE complex assembly (syntaxin-Munc18), driving rapid fusion of mucin granules with the apical membrane [109–111]. Simultaneously, Ca^2^⁺-activated TMEM16A furnishes Cl⁻ and water flux that hydrates the expelled mucin mesh, while KCa3.1 (IKCa1) hyperpolarises the membrane via K⁺ efflux, sustaining the electrochemical gradient for continued Ca^2^⁺ influx and secretion [112]. Persistent Ca^2+^ activates CaM, which feeds calcineurin-NFAT and CaMK/PKC pathways to up-regulate MUC5AC transcription in cooperation with EGFR-STAT6 inputs [100]. Major gel-forming mucins contain Ca^2^⁺-binding CysD domains that assist disulfide-mediated polymerisation; when SOCE- or Piezo1-driven Ca^2^⁺ flux is excessive, these domains promote hyper-polymerisation, yielding the viscous, tenacious plugs characteristic of asthma [105–107]. Thus, from initial GPCR triggering to final CysD cross-linking, Ca^2^⁺ acts as the central coordinator of mucin exocytosis, hydration and pathological gel formation (Figs. 2A, B).

### Calcium ion basis of AHR: the causal role of homeostasis disruption

#### Calcium homeostasis and airway smooth muscle contraction

In asthma, the development of AHR in ASM stems from two core mechanisms that operate independently yet can synergistically amplify each other: (1) Increased calcium load, referring to an abnormal elevation in [Ca^2^⁺]i, which provides a stronger chemical drive for contraction; and (2) Enhanced calcium sensitivity, resulting from the inhibition of myosin light chain phosphatase (MLCP) activity, which leads to hyperreactivity of the contractile apparatus to existing Ca^2^⁺ levels—even without a further increase in Ca^2^⁺ concentration—thereby generating greater contractile force. Clearly distinguishing between these two pathways is essential for understanding AHR and developing targeted therapeutic strategies.

In asthma, aberrant hyperreactivity of ASM cells is a core feature. As previously outlined, persistent inflammation mediated by immune cells characterizes the airways of asthmatic patients. Key inflammatory mediators (e.g., IL-13, TNF-α, IL-4, IL-5) promote the development of AHR by activating both ASM and epithelial cells. Calcium homeostasis plays a pivotal role in regulating ASM contraction. ASM contraction is critically dependent on an elevation in [Ca^2^⁺]i. This rise in [Ca^2^⁺]i originates from extracellular Ca^2^⁺ influx and release from intracellular stores. Calcium homeostasis is subsequently restored through Ca^2^⁺ reuptake into intracellular stores (e.g., sarcoplasmic reticulum, SR) and extrusion from the cell, processes in which mitochondria also play significant roles. In asthma, reduced expression of SERCA2 [113] is observed and associated with key pathological features such as airway remodeling and enhanced ASM contraction. Downregulation of SERCA2 disrupts intracellular calcium homeostasis, manifesting as elevated [Ca^2^⁺]i and impaired Ca^2^⁺ clearance kinetics. This dysregulation contributes to hypercontraction and potentiates the inflammatory milieu. Studies have revealed that conditioned medium derived from AECs of asthmatic patients subjected to mechanical compression exhibits a stronger potentiating effect on histamine-induced ASM contraction compared to controls, even at lower compression pressures. This finding suggests that epithelial cells in asthmatic patients may possess heightened sensitivity to mechanical stress or exhibit impaired protective functions [114].

#### Fundamentals of airway smooth muscle contraction and relaxation [115]

Contraction of ASM is primarily dependent on [Ca^2^⁺]i elevation.. Elevation of [Ca^2^⁺]i occurs following extracellular Ca^2^⁺ influx through VGCCs or receptor-operated Ca^2^⁺ channels (ROCCs). The increased Ca^2^⁺ binds to CaM, forming a Ca^2^⁺/CaM complex. This complex subsequently activates MLCK in the cytosol, which then phosphorylates the regulatory myosin light chain (MLC20). Phosphorylation of MLC20 governs the interaction between myosin and actin, promoting the formation of actin-myosin cross-bridges, ultimately leading to ASM contraction.

Relaxation of ASM relies on the dephosphorylation of MLC20 by myosin light-chain phosphatase (MLCP). MLCP dephosphorylates MLC20 by hydrolyzing its phosphate group, thereby restoring myosin to an inactive state. This terminates the binding between myosin and actin, resulting in muscle relaxation. The activity of MLCP is regulated by multiple signaling pathways, with ROCK being a major inhibitory factor. Under pathological conditions, such as asthma, activation of ROCK inhibits MLCP activity. This leads to sustained phosphorylation of MLC20, thereby maintaining or exacerbating the contracted state of the muscle (hypercontraction).

### Intrinsic calcium signaling abnormalities in ASMCs—basis for hypercontraction

#### Increased calcium load

In ASMCs of asthmatic patients, the elevation of [Ca^2^⁺]i arises through multiple mechanisms. First in human lung tissue sections from moderate-to-severe asthmatics (n = 8), Goriounova et al. (2023) reported significantly increased co-localization of Orai1 and STIM1, indicating enhanced Orai1 activity, particularly in epithelial and immune cells. This was associated with increased Orai1 aggregation, suggesting hyperactive SOCE-mediated calcium signaling in asthma, although Orai1 expression levels per se were not consistently upregulated [116, 117]. Second, intracellular Ca^2^⁺ release is augmented. Inflammatory mediators activate G protein-coupled receptors, increasing IP₃ production via phospholipase Cβ. Concurrently, elevated expression or sensitivity of IP₃ receptors may enhance Ca^2^⁺ release from the SR [118]. Furthermore, impaired Ca^2^⁺ clearance/reuptake capacity is a critical factor. Reduced expression or activity of sarco/endoplasmic reticulum Ca^2^⁺-ATPase (SERCA), particularly the SERCA2 isoform, along with direct inhibition of its function by inflammatory and oxidative stress byproducts, compromises Ca^2^⁺ resequestration into the SR. The activity of the plasma membrane Ca^2^⁺ ATPase (PMCA) and the Na⁺/Ca^2^⁺ exchanger (NCX) may also be suppressed [119]. Collectively, these defects prevent the efficient clearance of elevated [Ca^2^⁺]i, thereby sustaining the contracted state. Additionally, certain inflammatory milieus or oxidative stress may perturb ryanodine receptor (RyR) function, leading to enhanced CICR or abnormal spontaneous Ca^2^⁺ release events (e.g., Ca^2^⁺ sparks, Ca^2^⁺ waves), which can more rapidly initiate contraction [120, 121].

#### Enhanced calcium sensitivity

Acting in parallel with increased Ca^2+^ load, enhanced calcium sensitivity represents another independent and critical mechanism driving AHR. This process is predominantly mediated by activation of the RhoA/ROCK pathway. Inflammatory mediators associated with asthma serve as key activators of this signaling axis. Upon activation, ROCK phosphorylates and thereby inhibits the activity of myosin light chain phosphatase (MLCP) [122]. The loss of MLCP function impairs dephosphorylation of the phosphorylated myosin light chain (p-MLC20), resulting in sustained actin-myosin cross-bridge cycling and enabling greater contractile force generation even at identical levels of [Ca^2^⁺]i. Thus, the mechanism of enhanced calcium sensitivity acts to amplify the contractile signal initiated by increased calcium load.

#### Integrated mechanisms of calcium homeostasis disruption leading to AHR

Asthma exacerbations begin with allergens, viruses or pollutants that trigger epithelial danger signals and Th2-biased antigen presentation [123]. IL-4/IL-13 and IL-5 drive IgE-dependent mast-cell and eosinophil activation, releasing histamine, leukotrienes and PAF that act directly on ASM surface receptors [124]. These mediators rapidly elevate IP₃, open ryanodine receptors and hyper-activate STIM1/Orai1 SOCE, producing a sustained cytosolic Ca^2^⁺ surge that outlasts the acute stimulus [125]. Concurrently, ET-1 and cysteinyl-leukotrienes switch on RhoA/ROCK, phosphorylating MYPT1 and locking MLCP in an inactive state—this increases Ca^2^⁺ sensitivity without further global Ca^2^⁺ rise [126]. Furthermore, inflammation-generated oxidative stress inhibits SERCA function, resulting in delayed Ca^2^⁺ clearance [127]. Collectively, these alterations significantly enhance ASMC contractility in response to low-dose stimuli, manifesting as a reduced threshold for contraction, increased contraction amplitude, and prolonged contraction duration. This aberrant hyperresponsiveness ultimately culminates in the development of AHR. Epithelial necrosis and tight-junction loss expose sub-epithelial nerves and ASM to irritants, triggering neurogenic release of substance P and neurokinin A that further raise Ca^2^⁺ and potentiate contraction [123]. Compressed asthmatic epithelium releases ET-1, IL-33 and ATP at pressures innocuous to healthy controls, amplifying ASM Ca^2^⁺ flashes via Piezo1/TRPV4-P2Y pathways [61, 114, 128]. Thus, inflammation sets a high basal Ca^2^⁺ set-point, while mechanical deformation supplies intermittent Ca^2^⁺ spikes—together a self-amplifying vicious cycle. Persistent inflammation drives ASM hyperplasia, goblet-cell metaplasia and sub-epithelial fibrosis [129]. Hyperplastic ASMCs exhibit similar dysregulation of calcium signaling and enhanced contractility, further exacerbating AHR.

**Fig. 3:**
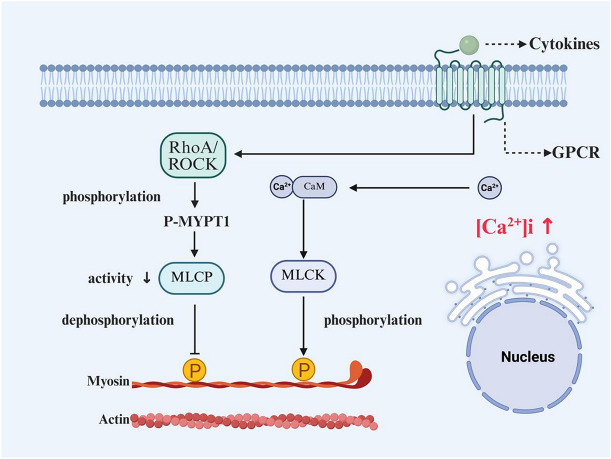
Dual calcium–calci-sensitization pathways in asthmatic airway smooth-muscle hyperresponsiveness. Inflammatory mediators trigger the GPCR → RhoA-ROCK axis; ROCK phosphorylates MYPT1, inhibits MLCP and increases Ca^2^⁺ sensitivity. Concurrently, PLC-IP₃-mediated intracellular Ca^2^⁺ release and mechanically induced extracellular Ca^2^⁺ influx (detailed in Fig. 1B) elevate [Ca^2^⁺]i; Ca^2^⁺/CaM activates MLCK, promoting myosin phosphorylation. The two arms synergistically enhance sustained Actin–Myosin cross-bridge cycling, leading to prolonged ASM contraction and airway hyperresponsiveness

ASMC hypercontraction, sustained amplification of inflammation, and epithelial injury mutually reinforce one another, establishing a self-perpetuating pathological cycle that collectively drives and perpetuates AHR. At the core, the chronic inflammatory milieu in asthma, characterized by the presence of Th2 cytokines and inflammatory mediators, constitutes the fundamental driver of disrupted calcium homeostasis across multiple airway cell types, particularly ASMCs. Regarding intrinsic alterations within ASMCs, augmented SOCE, primarily due to upregulated STIM1/Orai1 expression, serves as a major mechanism for excessive elevation of [Ca^2^⁺]i. Concomitant key alterations include enhanced Ca^2^⁺ release (increased IP₃ receptor [IP₃R] sensitivity and expression), impaired Ca^2^⁺ clearance/reuptake capacity (inhibition of SERCA, and markedly increased Ca^2^⁺ sensitivity (activation of the RhoA/ROCK pathway, which inhibits myosin light-chain phosphatase [MLCP] activity). Inflammatory cells undergo activation, degranulation, and cytokine secretion in a calcium signaling-dependent manner. The mediators released directly stimulate ASMC contraction and sustain the inflammatory cascade, thereby amplifying the pathological process. Concurrently, epithelial injury compromises barrier function, augmenting exposure to irritants and reducing the production of protective relaxant factors. This further exacerbates the pathological progression of asthma. (Fig. 3).

#### The central role of mechanical stress in asthmatic airway remodeling

Mechanical stress plays a pivotal role in airway remodeling in asthma, a concept robustly supported by multiple studies [130, 131]. Asthma is a chronic inflammatory airway disease. Airway remodeling denotes persistent structural alterations of the airway wall, encompassing epithelial injury, goblet cell hyperplasia, subepithelial thickening, ASM hyperplasia, and angiogenesis. Traditionally, airway remodeling was attributed predominantly to chronic inflammation. However, accumulating evidence underscores the critical contribution of mechanical stress to this process. Mechanical stress influences airway remodeling in asthma through multiple pathways, primarily targeting three key cell types: AECs, fibroblasts, and ASM cells.

#### Quantitative aspects of mechanotransduction in asthma

To reconcile disparate findings regarding cellular responses to mechanical stress, it is essential to consider the quantitative parameters of the applied stimuli. In vitro studies employ a range of mechanical inputs: compressive stresses typically range from 5 to 30 cmH₂O (~ 0.5 to ~ 3 kPa), while cyclic stretch regimens often use 5–20% elongation at frequencies of 0.1–0.3 Hz (mimicking normal breathing) to 0.5–1.0 Hz (simulating tachypnea). The duration of stimulation also varies significantly, from acute exposures (minutes to hours) probing immediate signaling events to chronic applications (days) modeling remodeling. This wide spectrum of magnitude, frequency, and duration likely underlies the context-dependent outcomes—such as proliferation versus apoptosis—observed in different studies. The following sections detail the effects of these quantified mechanical stresses on specific airway cell types. The physiological relevance of in vitro mechanical stimuli must be interpreted with caution, as parameters vary widely between studies, and only a subset may accurately recapitulate the stresses experienced during asthmatic bronchoconstriction.

#### Effects on AECs

Mechanical stress (e.g., compressive stress) activates epithelial cells, inducing the release of key mediators implicated in airway remodeling, such as TGF-β [132], ET-1 [133], and plasminogen activator [134]. It also upregulates the expression and secretion of cytokines and ECM components. For instance, mechanical stress induces upregulation MUC5AC, enhances expression of EGFR and its ligands, and stimulates production of matrix metalloproteinases (MMPs), specifically MMP-2 and MMP-9 [135–137]. Mechanical compression resulting from bronchospasm is sufficient to induce the production of ECM proteins within the airways, thereby potentially contributing to airway remodeling [138]. Furthermore, mechanical stress may promote the secretion of YKL-40 (a biomarker associated with airway remodeling) and tissue factor (which promotes angiogenesis) [139]. Cyclic mechanical stretch increases ROS generation in AECs and upregulates prostaglandin E₂ (PGE₂) synthesis [140]. PGE₂, in turn, promotes fibroblast proliferation and collagen production [141].

#### Effects on airway fibroblasts

Fibroblasts are key mechanosensitive cells that transduce mechanical signals into biological responses, particularly regarding the expression of ECM genes. Evidence indicates that Piezo1 plays a critical role in mechanical stretch-induced remodeling of lung fibroblasts. Piezo1 mediates mechanical stretch-induced fibroblast-to-myofibroblast transition (FMT) and ECM formation via activation of the extracellular signal-regulated kinase (ERK) signaling pathway and Ca^2^⁺ influx [142]. Mechanical stress can also upregulate the mRNA expression of versican, decorin, and procollagen.

However, the impact of mechanical stretch on cell proliferation remains controversial. Some studies indicate that mechanical stretch enhances fibroblast proliferation and collagen production [143], whereas others report an increase in apoptosis and a concomitant reduction in proliferation [144].

#### Effects on ASMCs

Bo Lan et al. demonstrated that mechanical compression of AECs can induce increased proliferation of ASMCs [114]. Additionally, Luo et al. found that high-amplitude stretch promotes ASMC migration but reduces cell adhesion [145]. These findings suggest that mechanical stretch may exacerbate airway remodeling in asthmatic patients by promoting ASMC proliferation and migration. Mechanical stretch can perturb ASMC calcium homeostasis, consequently affecting their contractile properties and function. Yang Yao et al. highlighted the critical role of mechanosensitive Piezo and TRP channels in the airways, implicating them in the regulation of ASM contraction and remodeling [128]. Mechanical stretch can also increase ASM stiffness and enhance contractile function [146]. In human asthmatic patients, ASMC apoptosis has been observed alongside increased ASM mass, and both parameters show a positive correlation with asthma severity [147]. Collectively, these data indicate that mechanical stretch likely influences airway remodeling by modulating multiple ASMC processes, including proliferation, migration, adhesion, calcium homeostasis, and apoptosis.

In summary, compressive stress generated by ASM contraction (e.g., during asthma attacks) and additional frictional forces imposed by AMH on the contracting airway wall act upon AECs, fibroblasts, and ASMCs themselves. This leads to aberrant activation of these cells and alterations in ECM composition. Ultimately, these changes drive structural alterations and fibrosis of the airways, representing a significant mechanism underlying airway remodeling.

### The pathological cascade driven by calcium signaling dysregulation in airway remodeling

Calcium signaling dysregulation plays a pivotal role in airway remodeling in asthma, involving a cascade of pathological events across multiple cellular and molecular levels. Aberrant regulation of intracellular Ca^2^⁺ profoundly impacts ASMC function, encompassing proliferation, migration, and contractile capacity. For instance, downregulation of SERCA2 elevates [Ca^2^⁺]i. This subsequently activates multiple signaling pathways, including the PTK2 (focal adhesion kinase, FAK)/NF-κB/calcineurin axis, promoting the transcription of cyclin D1 and driving ASMC proliferation [148]. Furthermore, enhanced activity of TRPC3 channels contributes to Ca^2^⁺ influx, further exacerbating airway remodeling [149].

Disturbances in calcium signaling are also closely associated with the damage and repair processes of AECs [79]. Damage to AECs releases various inflammatory mediators and growth factors, such as TGF-β1 and HB-EGF, which promote fibroblast proliferation and collagen deposition, thereby contributing to airway remodeling. Furthermore, aberrant calcium signaling may impair mitochondrial function, leading to mitochondrial Ca^2+^ overload, which subsequently affects cellular metabolism and apoptosis [150].

In ASMCs, dysregulated calcium signaling can also impact cellular contraction and proliferation by modulating the activity of CaM and calcium/calmodulin-dependent protein kinase II (CaMKII). For instance, activation of CaMKII promotes the expression of cell cycle-related genes, thereby enhancing ASMC proliferation. Additionally, abnormal calcium signaling may affect ASM contractility and remodeling potential by influencing the RhoA/Rho kinase pathway [122].

Calcium signaling disturbances may also exacerbate airway remodeling by modulating airway inflammatory responses. For example, aberrant calcium signaling can activate multiple inflammatory mediators, such as IL-4, IL-5, and IL-13, which promote both the proliferation of ASMCs and inflammatory reactions. Moreover, dysregulated calcium signaling may further modulate the airway immune milieu by influencing the release of TSLP and IL-33 [79].

In summary, disturbances in calcium signaling plays a central role in airway remodeling in asthma. This pathological cascade involves intricate interactions among ASMCs, AECs, and various inflammatory mediators. Understanding these mechanisms is crucial for the development of novel therapeutic strategies.

### Calcium signaling in non-type 2 and severe asthma endotypes

While this review has primarily focused on the well-characterized roles of calcium signaling in T2-high asthma, emerging evidence indicates that dysregulated calcium signaling is not confined to T2-high asthma but may rather serve as a universal amplifier of inflammation and airway dysfunction across all asthma phenotypes. This is particularly evident in severe asthma endotypes characterized by neutrophilic infiltration, an IL-17-high phenotype, and glucocorticoid (GC) resistance, where abnormal activation of Ca^2^⁺ channels is supported by a growing body of experimental and clinical data [79]。

Calcium signaling plays a pivotal role in IL-17-driven neutrophilic inflammation. IL-17 promotes neutrophilic infiltration, a core mechanism in severe asthma, by inducing the secretion of chemokines such as IL-8, CXCL1, and G-CSF from epithelial cells [151]. The IL-17 pathway acts synergistically with environmental exposures like cigarette smoke and ozone, which are potent inducers of oxidative stress. This oxidative milieu directly activates redox-sensitive, Ca^2^⁺-permeable channels, notably TRPV1 and TRPA1, on airway sensory nerves and epithelial cells, The study also found that CS/CSE could rapidly increase the intracellular calcium ion concentration of hASMC cells, which was mainly mediated by TRPA1 channel-mediated calcium influx, and further led to MLC phosphorylation, which was closely related to the contractility of ASM [19, 30, 152]. The ensuing sustained Ca^2^⁺ influx, in turn, drives the production of potent neutrophil chemoattractants like IL-8/CXCL8, thereby reinforcing a pro-inflammatory feedback loop that exacerbates neutrophilic inflammation.Traditional Chinese herbal medicine, with its multi-target, low-toxicity, and high bioactivity characteristics, has great potential in treating asthma by modulating TRPV1/TRPA1 channels [153].

Furthermore, aberrant calcium signaling represents a compelling mechanistic candidate underlying glucocorticoid resistance. Sustained Ca^2^⁺ influx through channels such as TRPV1 and Piezo1 leads to the activation of NF-κB. This transcription factor not only drives steroid-insensitive inflammation but also engages in cross-antagonism with the GR—for instance, by competing for binding sites or upregulating the dominant-negative GR-β isoform—thereby impairing the anti-inflammatory effects of glucocorticoids [154, 155]. Consequently, this Ca^2^⁺-mediated, hyperactive pro-inflammatory state can establish a molecular environment inherently less responsive to conventional corticosteroid therapy.

Collectively, these insights advocate for the recognition of "calcium endotypes" as a novel stratification paradigm. This concept posits that a subset of asthmatic patients, irrespective of their T2 status, may exhibit a pathologically hyperactive calcium signaling network as a core driver of their disease. Identifying such patients through biomarkers related to Ca^2^⁺ pathway activity (e.g., expression levels of specific channels, in vivo Ca^2^⁺ imaging) could delineate a population most likely to benefit from Ca^2^⁺-targeted therapies. Therefore, targeting key calcium nodes may offer a groundbreaking therapeutic strategy for severe, steroid-refractory asthma, potentially restoring treatment response where current anti-inflammatory regimens have failed.

## Discussion

This review synthesizes recent evidence to propose that dysregulation of calcium homeostasis is not merely an epiphenomenon of asthmatic inflammation but rather a core driver underpinning the entire disease process. Mechanical factors—be it airway constriction, mucus plugging, or external airflow shear stress—can activate mechanosensitive channels (e.g., Piezo1, TRPV1, TRPA1), triggering intracellular Ca^2^⁺ influx. This effectively "translates" physical stimuli into pathological signals, amplifying inflammatory responses, disrupting the epithelial barrier, and promoting tissue remodeling. AECs are particularly vulnerable in this process. Mechanical stress synergizes with Th2 cytokines (notably IL-13) to induce sustained Ca^2^⁺ overload. This activates calpain, leading to the degradation of tight junction proteins (occludin, E-cadherin). Concurrently, NFAT-dependent transcription upregulates the expression of mucins (MUC5AC, MUC5B) and pro-inflammatory mediators (TSLP, IL-25). Furthermore, the loss of ZO-1 and cytoskeletal disarray further impair the epithelium’s self-repair capacity. This establishes a vicious cycle: barrier disruption → allergen penetration → further amplification of calcium signaling. Concurrently, ASMCs exhibit intrinsic Ca^2^⁺ hypersensitivity. SOCE is hyperactivated due to upregulated STIM1/Orai1, while impaired function of the SERCA delays Ca^2^⁺ clearance. Additionally, activation of the RhoA-ROCK pathway heightens the sensitivity of the contractile apparatus to Ca^2^⁺. Consequently, even low-level stimuli can trigger intense, sustained contraction. Moreover, mechanical stretch, acting via Piezo1, further promotes ECM secretion, directly linking hyperresponsiveness to airway remodeling. These findings indicate that current anti-inflammatory strategies, primarily based on corticosteroids and biologics, fail to adequately interrupt the mechanically driven pathological circuits. Future precision interventions could consider a triple-targeting strategy: (a) Piezo1 inhibitors (e.g., GsMTx-4) to block mechano-chemical signal transduction; (b) SOCE inhibitors (e.g., CM2489) to suppress excessive ASM contraction and mucus secretion; and (c) a combination of anti-IL-13 biologics with calcium channel modulators to synergistically restore epithelial barrier function. However, clinical translation requires addressing three critical challenges: First, delineating the spatiotemporal dynamics of calcium signaling in in vivo asthma models. Second, defining the cell type-specific roles (e.g., in ILC2s, ASM, epithelial cells) of Piezo1/TRP channels. Third, identifying reliable biomarkers to stratify patient subpopulations with "hyperactive calcium signaling pathways." Elucidating these aspects holds promise for advancing calcium homeostasis modulation from mechanistic investigation towards a novel paradigm for personalized asthma therapy. The clinical translation prospects, candidate agents, and associated challenges for targeting these calcium signaling nodes are systematically outlined in Table 2.

**Table 2:** Clinical transformation prospects and challenges of calcium signaling node targeted therapy in asthma

Target of therapy	Representative candidate drug/tools	Current development phase	Major potential risks and challenges
PIEZO1	GsMTx-4	Tool peptide	Lack of specificity (may affect other mechanically sensitive channels); poor pharmacokinetics of peptide drugs (low oral bioavailability, short half-life); potential cardiovascular side effects of systemic administration
SOCE (STIM/Orai)	CM2489	Early-phase clinical/preclinical	Immunosuppressive risk (because the function of immune cells such as T cells is highly dependent on SOCE); may affect the calcium homeostasis of other non-target cells
TMEM16A (ANO1)	Niclosamide/Nitazoxanide	Drug repurposing/new indication exploration	It may disrupt epithelial transport and normal mucous ciliary clearance function, resulting in dry eye, dry mouth and other side effects Lipophilicity and Slow Onset of Action
LTCC	Bipyridines (e.g., nifedipine)	Approved—off-label use	Cardiovascular side effects (hypotension, tachycardia, peripheral edema) limit its high dose use in asthma
TRP channel (TRPV1/A1)	Multiple antagonists	Clinical development halted/preclinical	Early clinical trials were discontinued due to serious off-target effects such as thermoregulatory dysfunction (leading to high fever risk)

This table systematically summarizes the clinical drug development status of the targeted molecules discussed in this study. The data reveals that no target-specific drugs have been approved for clinical treatment of asthma, and single-target therapies cannot address all ion channel types. Given our investigation into mechanical stress-induced calcium homeostasis imbalance, along with studies on how airway mucus and ECM amplify mechanical stress to activate related ion channels beyond inflammation, this research provides clinicians with a fresh perspective: the critical role of airway mucus in asthma pathogenesis

### Interactions with current asthma therapies: calcium nodes as secondary targets

β₂-Adrenergic agonists are still the first choice for bronchodilation, but frequent high-dose use can lead to desensitization of β2-AR and reduce efficacy, while its secondary effects on calcium regulation have not been fully paid attention. β2-adrenergic agonists induce airway relaxation by reducing the Ca^2^⁺ oscillation frequency of ASMCs through the cAMP/PKA/IP₃R pathway and decreasing Ca^2^⁺ sensitivity via dephosphorylation of MLC20. Formoterol demonstrates superior efficacy to salbutamol in both aspects, with a longer duration of action in human airways [156]. In addition, bitter-tasting compounds relax ASMCs by binding to taste receptor type 2 (TAS2R) in a way similar to traditional bronchodilators [157]. In alveolar epithelial cells (AECs), salbutamol regulates the abundance of Na,K-ATPase on AECs surface via SOCE channels, thereby influencing critical physiological processes of pulmonary fluid clearance. This "double-edged sword" of calcium signaling manifests as moderate SOCE promoting Na,K-ATPase recruitment, while excessive SOCE may lead to endocytosis [158]. Inhaled corticosteroids/long-acting β2 receptor agonists (ICS/LABA) can enhance serum Stanniocalcin-1 (STC1) levels in asthmatic patients. STC1 (particularly exogenous STC1) inhibits Ca^2^⁺ influx and MLC phosphorylation by suppressing STIM1-mediated SOCE, thereby reducing ASM contractility [159]. Similarly, ICS indirectly dampen SOCE by down-regulating STIM1/Orai1 transcription via NF-κB inhibition, an effect reversed in steroid-resistant patients with sustained NF-κB activation [79]. These data suggest that current therapies already modulate Ca^2^⁺ nodes, but inefficiently in severe or steroid-refractory disease, leaving a therapeutic gap for direct Ca^2^⁺-targeted adjuncts.

Biologics targeting IL-4/IL-13 (dupilumab, tralokinumab) effectively suppress Type-2 inflammation, yet fail to normalize TMEM16A expression in airway epithelia of severe asthmatics [160]. Since IL-13 → STAT6 → TMEM16A axis is a major driver of Ca^2^⁺-dependent mucus hypersecretion, residual MUC5AC production persists despite biologic therapy [161]. Conversely, IL-5 blockade (mepolizumab) reduces eosinophilia but does not inhibit Piezo1-mediated mechano-Ca^2^⁺ signaling, leaving mechanical stress → Ca^2^⁺ → AHR circuits intact [162]. These observations underscore the need for combination strategies that pair biologics with Ca^2^⁺-targeted agents to close the mechano-chemical signaling loop that current anti-inflammatory regimens leave unaddressed.

## Conclusion

In summary, dysregulation of calcium homeostasis serves not only as a "signaling transducer" between mechanical forces and inflammation, but also as a convergent pathway underlying asthma heterogeneity. Consequently, interventions targeting calcium signaling pathways can evolve from "single-factor blockade" to "remodeling the microenvironmental homeostasis," offering a precision strategy for refractory asthma. Future research must integrate calcium imaging with multi-omics technologies to establish individualized "calcium endophenotypes," thereby advancing asthma management from empiric therapy towards mechanism-guided precision medicine.

## Data Availability

No datasets were generated or analysed during the current study.

## Declarations

### Declaration on the use of generative AI tools in the writing process

During the preparation of this work, the author(s) used a generative AI tool to improve the readability and language of the manuscript, namely for language polishing. After using this tool, the author(s) reviewed and edited the content as necessary and take(s) full responsibility for the content of the published article.
